# Technology Considerations for Enabling eSource in Clinical Research: Industry Perspective

**DOI:** 10.1007/s43441-020-00132-4

**Published:** 2020-03-11

**Authors:** Donald G. Jennings, Amy Nordo, Aruna Vattikola, Jesper Kjaer

**Affiliations:** 1 Digital Health, Eli Lilly and Company, MC/525/02, Indianapolis, IN 46285 USA; 2 Clinical Trials Solutions Pfizer, Inc., Groton, CT USA; 3 Novartis Business Technology Services, East Hanover, NJ USA; 4 Novo Nordisk A/S, Bagsværd, Denmark

**Keywords:** Architecture, Digital, TransCelerate, IoT, Big data, Analytics

## Abstract

**Background:**

The technological complexities and broad operational scope of eSource impede coordinated, inter-organizational action on advancing at-scale solutions.

**Methods:**

We introduce an architectural framework for articulating technological considerations across organizations. The architecture neither implies nor endorses solution implementations; rather, it proposes solution functionality based upon principles and good clinical practices.

**Results:**

Key technology considerations include patterns of anticipated use, implications to the current state of clinical trial operations, and the need for new technologies (i.e., IoT, Big Data, Predictive Analytics).

**Conclusion:**

Technology considerations drive implications beyond technology—influencing regulatory, process, and ethical realms of clinical research.

**Electronic supplementary material:**

The online version of this article (10.1007/s43441-020-00132-4) contains supplementary material, which is available to authorized users.

## Background

Adoption of digital technologies into clinical research simultaneously disrupts and transforms how pharmaceutical companies (Sponsors) approach development of new therapies. It drives innovation, provides for planning and execution of higher value trials, drives discovery of correlations between digital signals and biological events, and has the potential to personalize therapeutic health outcomes.

Technology professionals should understand how digital methodologies disrupt current clinical research models and respond with solutions that address the new needs while simultaneously supporting global regulatory expectations, data integrity, and security and privacy concerns.

eSource is the digital vanguard of clinical research. Simply stated, eSource is *data initially recorded in electronic format* [1]. Since 2010, many regulatory authorities have either expressed interest in, or provided written guidance on, their expectations for use of eSource in clinical trials [1–5]. eSource is a timely topic, but the disruptions and associated technological implications have not been widely discussed.

eSource brings four significant technology disruptions into the clinical research domain.

- It moves clinical research into the realm of *Big Data*—with potential data volumes that far surpass those associated with main-line clinical trials.
- It builds an interoperability bridge with the Healthcare IT domain, allowing Sponsors to coordinate trial data with electronic health records (EHR) and improve the power of clinical research analysis.
- It drives the need for algorithmic data interpretation and reduction to transform raw digital signals into clinically meaningful, data-driven results.
- It requires use of internet-scale techniques for collection of coordinated time-series data from sensors, wearables, and mobile devices.

These disruptions unlock new opportunities to improve drug development effectiveness and usher in new classes of digitally enabled healthcare therapies. They also create a new class of challenges for the *Stakeholder* community—i.e., *trial sites, technology vendors, standards organizations, regulatory authorities, payers, ethics boards, healthcare professionals (HCPs), and Sponsors.* The challenges span the space of trial design, data collection and exchange, pharmacovigilance, data security and privacy, workforce skills, resource allocation, and regulatory expectations [6].

Previous TransCelerate publications [7] identify technology maturity as a significant factor obstructing eSource adoption within clinical research. An equally important obstruction is Stakeholder discord on the set of technologies needed to implement eSource at-scale.

**Diagram 1.**
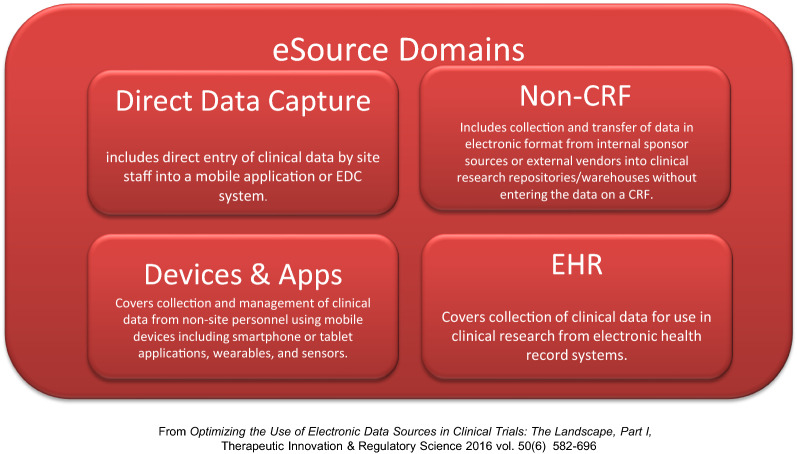
The Four Modalities of eSource.

The broad operational scope of eSource also complicates the goal of Stakeholder alignment. TransCelerate defines four eSource modalities: [7] *Direct Data Capture, Non*-*CRF, Devices & Apps,* and *EHR* (see Diagram 1). Stakeholders often speak to modality-specific use cases most relevant to their purview, without considering implications of use cases arising from the other modalities. This impedes Stakeholder alignment on a holistic path forward for eSource adoption, especially from the technology perspective.

In summary, the clinical research community faces challenges on the adoption of eSource arising from the associated technological complexities and broad operational scope. Coordinated Stakeholder action depends upon articulating the diverse perspectives with a common conceptual framework to build technological expectation alignment across the clinical research community.

To address these challenges, we posit an eSource Logical Architecture as the basis of a common conceptual framework. It elucidates key eSource technology considerations of data collection and processing systems, where such considerations require a common Stakeholder understanding and response. Using the Logical Architecture as a guide, the paper discusses potential patterns of use, identifies new technology capabilities and extrapolates the implications—i.e., disruptions and opportunities—to the state of clinical research.

## Methodology

Over the course of 2018, subject matter experts from TransCelerate Biopharma (TCB) member companies collaborated on a logical-level architecture, based upon previous TCB reference architecture work and the experience gained from internal efforts at the TCB member pharmaceutical companies. The resulting Logical Architecture provides a conveyance for Stakeholder discussion and evaluation of eSource technology considerations.

Logical architectures strive to provide as much detail as possible for resulting solutions without constraining those solutions to particular designs, environments, vendors, or technologies. This is in contrast to conceptual architectures, which explain what solutions should do in terms non-technical stakeholders can understand, and physical architectures which provide enough detail to implement and deploy resulting solutions.

The Logical Architecture conveys technology considerations, asserted functionality, and patterns of use without mandating specific solution implementations. The intent is to spur contemplation and discussion rather than supply recipes for solution construction. The completeness and absolute correctness of the Logical Architecture at this juncture is less important than the discussion it generates, the implications it asserts and the degree of alignment it drives.

## Logical Architecture

**Diagram 2.**
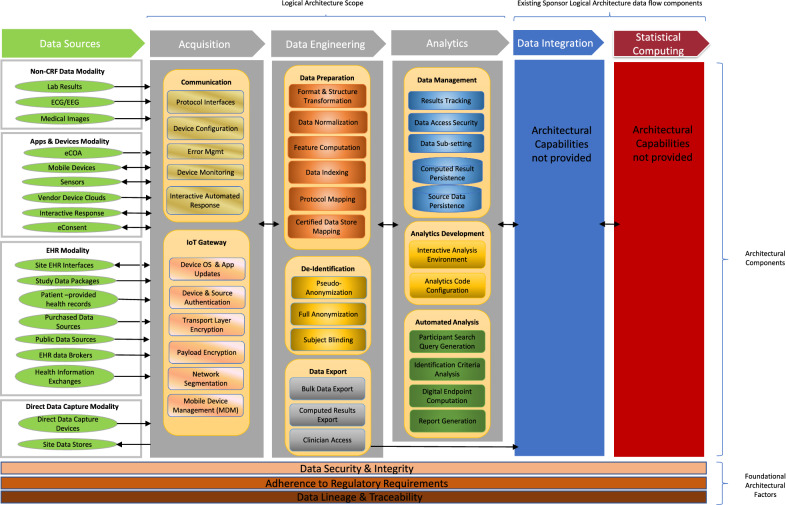
Logical Architecture.

The Logical Architecture (see Diagram 2) provides the framework for characterizing relevant technology considerations of eSource adoption. Data generally flow from left-to-right in the diagram—from clinical data sources to Sponsor data systems—but counter-flows from right-to-left are anticipated to implement specific patterns of use.

The six columns in Diagram 2—Data Sources, Acquisition, Data Engineering, Analytics, Data Integration, Statistical Computing—comprise the significant clinical data flow architectural components. The Acquisition, Data Engineering, and Analytics columns (in gray) encapsulate a broad class of new functionality relevant for digital data collection and correlation of digital signals with biological events. The Data Integration and Statistical Computing columns (blue and red) represent existing clinical data flow architecture within Sponsor data management systems and are not discussed further. Functionality used by the data sources (green) is likewise not discussed further.

### Acquisition

Main-line clinical trials utilize EDC (electronic data capture) systems to acquire data at trial sites and exchange it with Sponsors. EDC systems digitize the traditional CRF (case report form) process where clinicians record discrete data values on a per-patient basis during site visits. Digital data collection—aside from the *Direct Data Capture* modality—does not conform to EDC architectural principles for three key reasons: data collection can be continuous (e.g., time-series values), data might be un-mappable to CRF fields because it lacks self-evident clinical significance (e.g., “raw” digital signals), and incoming data volumes may exceed the EDC system storage capacity.

The general digital data acquisition scenario anticipates distributed networked devices engaged in autonomous communication with one or more centralized data aggregation agents: i.e., an Internet of Things (IoT) pattern. The *Things* in this scenario are the networked devices—ranging from personal health mobile devices, environmental sensors, central lab data feeds and institutional EHR systems. The acquired digital data can be biometric, behavioral, or environmental in nature.

### Data Engineering

Automated acquisition of continuous time-series data (biometric, behavioral, environmental) differs from acquisition of discrete data values in several ways. One key difference is the need for data integration agility if inconsistencies are detected in data streams, necessitating tradeoffs between system validation integrity and reaction times. The possible need for on-the-fly data de-identification and clinician access to near real-time summarized data also create differences in the technical approach.

Data Engineering incorporates agile and flexible transformation into the Logical Architecture, converting acquired data streams into common data structures with normalized units and appropriately de-identified/anonymized values. It provides access points for adding new analytics or transformation logic into the data flow with low disruption to existing components. It is also supports near real-time data stream access for implementation of HCP appropriate summarized data visualizations.

### Analytics

Main-line clinical trials typically perform data analysis as the last step: i.e., during statistical end point analysis. Data flows from the source (trial sites) to Sponsor statistical computing environments without addition or deletion, except for well-controlled corrections applied in the data review process.

eSource introduces data analysis via analytics throughout the trial lifecycle to reduce, interpret and clinically surface the incoming digital signals. This implies the Logical Architecture provide raw digital data persistence and analytics pre-processing prior to data ingestion by existing Sponsor clinical data management systems. The analytics can be exploratory or validated in nature. In the case of exploratory analytics, researchers attempt to discover correlations between digital signals and biological events. In the case of validated analytics Sponsors use known correlations that have been scientifically confirmed and accepted by Regulatory authorities to reduce digital signals into clinically relevant results.

### Architectural Factors

The three bottom rows of Diagram 2 represent factors that should guide eSource solution development for use in clinical research. They do not trace to classes of functionality; rather, they guide and constrain implementation of functionality.

- **Data Security and Integrity.** Clinical data, even when de-identified, may retain patient personal information attributes, and these data often are used in analysis to support the safety and efficacy of investigational medical therapies. Patients, HCPs, ethics boards and regulatory authorities must retain trust in the data for clinical research to function. Therefore, solutions must include data security and integrity considerations into their development.
- **Adherence to Regulatory Requirements.** Regulatory authorities throughout the world place requirements upon computer information systems used in clinical research. While many of these requirements share common attributes with professional software development expectations, some result in unique constraints upon how clinical research data systems should function. Therefore, solution providers must understand how these requirements affect implementation decisions.
- **Data Lineage and Traceability.** A key factor of data quality assurance involves the tracking of changes to data content—i.e., additions or deletions, who made changes and why—with un-alterable audit trails. Tracing the lifecycle of data records from data source through to analysis and reported outcomes is a necessary condition applied to all stages of implementation.

### Guiding Architectural Principles

The Logical Architecture is based upon five guiding principles that provide self-evident, *all things equal,* statements of truth. These principles are not meant as absolutes—organizational, resource or evolving regulatory constraints could alter the relevance of these principles to specific implementations. See Appendix A (supplementary material) for a full discourse on the architectural principles.

#### Limited Rework

Existing sponsor data flow architecture is not replaced in support of eSource, but rather augmented with new architectural capabilities.

#### Trial Site–Data Acquisition Decoupling

Regulatory considerations mandate that trial sites maintain the certified systems of record for trial data [8, 9]. In some scenarios, this tenant can be violated because data acquisition occurs outside of trial site control. eSource Solutions must account for this fact while simultaneously recognizing that trial sites maintain certified copies of the data.

#### Data Persistence and Computation Co-location

Data acquisition from multivariate, time-series data sources can generate volumes in the tera-byte to peta-byte range per study. Architectural decisions that improve data access and availability at the point of analysis improve computational cycle efficiency and, indirectly, the speed of clinical insights.

#### Clinical Data Relevance

eSource potentially generates data that lacks inferable *self*-*evident* clinical relevance. This leads to a distinction between eSource data with a priori intrinsic clinical relevance (e.g., blood pressure) vs. data whose clinical relevance requires algorithmic interpretation (e.g., raw digital signals).

#### Multi-use

The velocity of eSource adoption is in part governed by the ease study teams can integrate eSource use into study protocols. Architectures that reduce study team up front effort and implementation risk are favored. The easier the capability is to use, the more likely it will be used.

## Results

The Logical Architecture asserts a common conceptual framework for sharing Stakeholder perspectives, similar to use of a common coordinate system to share physical locations. The results below leverage the framework to articulate three technology considerations: new technologies, anticipated patterns of use, and implications to existing clinical research practices.

### New Technologies

Architectures do not mandate specific technologies, but they do prescribe boundary conditions: i.e., the available capabilities that solutions should use for development. In this regard, the Logical Architecture identifies three new technology capabilities for implementation of eSource solutions.

#### Internet of Things (IoT)

IoT represents networks of dedicated physical objects (things) that autonomously communicate their internal states as they sense and interact with external environments. This autonomous communication enables synchronized event capture across patient populations and data systems to derive end-user behavior, take preventive action, or augment business processes. Specifically for eSource, IoT enables at-scale use of network-connected device fleets for the acquisition of trial subject data and continuous patient health monitoring.

#### Big Data

Big Data is a set of techniques and associated technologies to analyze, systematically extract information from, or otherwise deal with data sets that are too large or complex for use in traditional data processing applications. This capability enables eSource to manage and analyze the large data sets resulting from continuous acquisition of time-series digital signals.

#### Predictive Analytics

Predictive analytics encompasses a variety of statistical techniques—e.g. data mining, predictive modeling, machine learning—which analyze current and historical facts across data sets to make predictions about future or otherwise unknown events. In addition to making statistical predictions between two effects, this class of analytics also allows for use of correlations between multiple variables to detect effects between events not present in single relationships. In the case of eSource, predictive analytics enable automated transformation of raw digital signals into clinically relevant data results, potential detection of trial subject health issues, and contextual combination of disparate data sources into merged data sets (e.g., data from EHR, EDC, and digital device data).

### Patterns of Use

When mapped to the Logical Architecture, the four eSource Modalities lead to twelve common *patterns of use.* A brief discussion of each pattern is provided below. See Appendix B (supplementary material) for a full discussion of the patterns.

#### Pattern 1: EHR Use in Clinical Trials

Direct site EHR to Sponsor clinical data exchange during clinical trials that eliminates double data entry (EHR and EDC) and data transcription errors.

#### Pattern 2: EHR Use in Real World Evidence (RWE) Studies

Direct EHR access by RWE teams to reduce startup study costs and expand dataset availability.

#### Pattern 3: Protocol-Based EHR Programming

Pre-populating site EHRs with study participant schedule of events to reduce manual effort and reduce transcription errors.

#### Pattern 4: EHR Patient Recruitment

Using site EHR data to identify potential study participant pools, using appropriate patient identity and privacy safeguards.

#### Pattern 5: eConsent

Use of electronic Informed consent techniques via mobile devices to lessen patient burden.

#### Pattern 6: App and Device Clouds

Use of cloud-based data aggregation points to encapsulate mobile device management and communication, alleviating the need for direct device support.

#### Pattern 7: Novel App and Devices

Implementing custom-built mobile device communication capability when pre-existing data aggregation points do not exist (e.g., experimental devices, custom sensors).

#### Pattern 8: Patient Registration and Device Setup

Using automated mobile device setup and direct-to-patient device distribution, removing the need for participants trial site visits to receive and setup mobile devices.

#### Pattern 9: Non-CRF Data Source Exchange

Flowing Non-CRF data generated by 3rd parities (e.g., central labs) into Sponsor data systems via automated data exchange.

#### Pattern 10: Capture of CRF Data Using Mobile Devices

Collecting CRF data at trial sites using mobile devices to eliminate clinician double data entry (e.g., site data system and Sponsor EDC) without limiting the ability of clinicians to record and maintain non-protocol mandated information [11]; for example, transmitting mobile device telemetry to site data stores prior to filtering/mapping and de-identifying the data values for Sponsor consumption [12].

#### Pattern 11: Exploratory and Analytics Development

Developing predictive analytics that discover and, subsequently, encapsulate correlations between digital signals and biological events.

#### Pattern 12: Synthetic Control Arms

Modeling study placebo arms with previously collected information to potentially improve patient care and study efficiency [13, 14].

### Implications

The Logical Architecture exposes technology-driven implications for clinical research. These implications could affect how Stakeholders utilize eSource technologies, or how clinical research evolves to meet the implications. The topics listed below illustrate some potential implications. A full discourse on the implications can be found in Appendix C (supplementary material).

#### Acquiring Data Across National Boundaries

The networked nature of digital data acquisition and persistence creates potential situations where data are gathered from patients or sites in country A from a centralized system physically instantiated in country B.

#### Adverse Events

Digital devices can generate constant and voluminous streams of patient health telemetry. If telemetry should indicate a patient Adverse Event (AE) or other health issue, will the system acquiring the telemetry be able to detect it? And if so, how will the acquiring system notify HCPs to intervene?

#### Data Review

Good Clinical Practice calls for Sponsors to review clinical data for inconsistences and ask trial sites for clarification/correction of unexpected data values. For the Devices and Apps modality, this practice becomes a practical impossibility due to the data volumes and complexities of digital signal inspection.

#### Analytics Validation

The data volume and complexity necessitate use of automated analytics to summarize and process the acquired data. These analytics occupy a new niche in the clinical data flow from existing statistical techniques, i.e., these analytics can affect trial operations as well as decisions made by HCPs in patient care.

#### Data Validation and Integrity

The nature of digital data acquisition and transformation introduces complexities into traditional clinical research data quality safeguards. Four specific use cases highlight this new implication to clinical research: differing EHR data conventions, safeguarding site EHR data from unintentional but improper Sponsor access, agile system updates vs. principles of traditional system validation, and trust of eSource data origination.

#### HCP Access to Digital Data Streams

Devices and Apps data streams provide a potentially useful patient monitoring capability; however, these data are generally unsuitable for HCP use in its raw form. Data summarizations via analytics potentially enable HCP data usability, but might also introduce new patient risks if HCPs cannot access or correctly interpret the results.

#### Systems of Record

With use of eSource, trial sites do not necessarily acquire the digital data. When data are not directly acquired by trial sites the system of record principle becomes difficult to maintain.

#### Primary Investigator Signoff

In main-line clinical trials that use EDC systems for data acquisition, the process of site Primary Investigator (PI) data approval is a straightforward activity, i.e., the PI reviews and electronically signs each eCRF sent to the Sponsor to assure its validity. As eSource data do not generally flow through an EDC system, the existing process of PI data approval cannot be duplicated.

## Discussion

### Personal Data Exchanges and Personal Health Records

Every patient may eventually be their own eSource data provider. Personal Health Records (PHR) and Personal Data Exchange (PDE) mechanisms allow individuals to capture, manage, and share their own health data. Only *early*-*adaptor* clinical research efforts have leveraged PHR, and PDEs are just now entering widespread use. The Logical Architecture includes consideration of PHR, but the practical implementations needed for at-scale use are not yet understood.

PHR information content is similar to an EHR, but they are managed directly by patients [15]. In 2006, the National Committee on Vital and Health Statistics (US Health and Human Services) published a report and recommendations for Personal Health Records [10]. The concept of PDEs generalizes PHR to all types of personal online data. PDEs are technology platforms that enable individuals to own their personal data and form the kernel of a rapidly emerging Personal Data Economy [16].

### Importance of Standards

Practical system-to-system clinical data exchange requires wide adoption of data standards and the ability to translate between them. Three standards in particular—HL7 FHIR, CDISC CDASH, and OMOP CDM—are key for at-scale system interoperability.

The HL7 Fast Healthcare Interoperability Resources (FHIR) standard promises to revolutionize healthcare data accessibility. FHIR defines common data structures and vocabularies as well as a scalable system-to-system exchange protocol. Healthcare community and IT vendor momentum behind FHIR adoption are strong, especially in the US; however, several important FHIR resource types (e.g., ResearchStudy, ResearchSubject) lack wide adoption, as does a standard process for trial site-to-Sponsor exchange. Like-minded sites and Sponsors, such as the SCDM eSource Implementation Consortium [17], have begun to tackle the logistical challenges. The need for wider FHIR resource adoption remains a topic for further consideration.

The desired target-state of standards interoperability foresees generalized FHIR resource mappings to the Clinical Data Interchange Standards Consortium (CDISC) and Observational Medical Outcomes Partnership (OMOP) family of standards. FHIR, CDISC, and OMOP encapsulate conceptually similar data domain models, but the specifics of mapping between them are an enormous undertaking. A number of initial efforts in this space are in progress [18, 19]. Stakeholders should recognize the foundational importance of this work to promoting healthcare data use in clinical research.

### Data Reuse

The US National Institutes of Health defines Data Reuse as a “*concept that involves using research data for a research activity or purpose other than that for which it was originally intended*. ” [20] eSource methodologies accelerate data reuse from patient health records and previous study data sets by reducing data acquisition friction and facilitating data set merging—making data usable for alternate purposes.

The advantages of clinical data reuse range from predictive analytics development to RWE studies and Phase I/II study synthetic arm construction (see the section on *Patterns of Use*). Each of these uses potentially accelerate trial execution speed, lower study costs and provide improved patient care outcomes. But the advantages come with associated challenges: e.g., informed consent, data quality, patient privacy, data ownership, and intellectual property concerns. [21]

## Conclusion

The digital paradigm simultaneously disrupts and transforms how Sponsors approach therapeutic development. Using the eSource Logical Architecture as a basis to quantify implications, we find that the disruptions and transformations affect the interests of all Stakeholders—*i.e., trial sites, technology vendors, standards organizations, regulatory authorities, payers, ethics boards, healthcare professionals (HCPs), and Sponsors*. The technology considerations make it clear that Stakeholders must act in unison along several fronts to achieve higher value clinical trials, new medical insights, and personalized health outcomes.

Previous survey results show that Sponsors embrace use of eSource, and Stakeholders want to modernize how the clinical research community develops supporting evidence for new drugs [1]. Regulatory authorities are now considering the complexities of eSource deployment and providing official guidance on topics such as EHR use [22]. The collective intent to ramp up eSource use is evident and the requisite foundational technology components, as depicted in the Logical Architecture, exist today.

All fundamental ingredients to integrate eSource into clinical research and gain the benefits of at-scale use are currently, or soon to be, available. The significant challenge facing Stakeholders is how and when to combine the ingredients and tackle the resulting regulatory, process, and ethical consequences.

## Electronic supplementary material

Below is the link to the electronic supplementary material.

Supplementary material 1 (DOCX 17 kb)

Supplementary material 2 (DOCX 712 kb)

Supplementary material 3 (DOCX 34 kb)

Supplementary material 4 (XLSX 13 kb)
