# Supplementary material for: Technology Considerations for Enabling eSource in Clinical Research: Industry Perspective
Source: Ther Innov Regul Sci. 2020 Mar 11;54(5):1166–74. doi: 10.1007/s43441-020-00132-4 (PMC7458892; doi:10.1007/s43441-020-00132-4)
Supplement: Supplementary file 1 — Supplementary material 1 (DOCX 17 kb) [file 43441_2020_132_MOESM1_ESM.docx]

Technology Considerations for enabling eSource in Clinical Research:

Industry Perspective

# Appendix A: Guiding Architectural Principles

The Logical Architecture is built from the guiding principles below. These principles are not necessarily inclusive of all relevant precepts and could be superseded by other considerations – e.g., organizational, regulatory, and financial – for any eSource solution implementation. They should be taken as *all things equal* statements of validity, rather than absolutes.

## Limited Rework

Existing sponsor data flow architecture is not replaced in support of eSource, but rather augmented with new capabilities. eSource is disruptive in that it affects how Stakeholders conduct clinical research; but eSource should not disrupt to the point that it forces Stakeholders to re-implement all existing processes and infrastructure. Re-work of existing systems must be minimized for the following reasons.

- Stakeholders spent decades arriving at the processes now in use. These processes often carefully balance patient safety with practicality and were arrived at through great expense.
- Continued uptake of eSource use in clinical research depends upon control of risk and cost. The net benefits of eSource must exceed its cost and risk by acceptable margins. All disruption to existing clinical data flow infrastructure and process introduces risk and cost. The fewer disruptions to existing infrastructure and process the better, all things equal.

## Trial Site-Data Acquisition Decoupling

Regulatory considerations mandate that trial sites maintain the clinical data certified systems-of-record^1,2^. In most mainline trial scenarios this consideration is straightforward to implement because site data systems perform initial data acquisition. For *Non-CRF*, *Devices & Apps* and some *Direct Data Capture* modality use cases this practical convenience is violated because data acquisition occurs outside of trial site control. Examples:

- Non-CRF modality: data samples analyzed at Central Labs flow into Sponsor clinical data systems without first passing through the trial sites.
- Devices and Apps: digital signals generated on mobile devices and sensors co-located with patients upload to cloud-based data aggregation agents that are neither supplied by, nor necessarily operated by, the trial sites.
- Direct Data Capture: sponsor-supplied mobile data collection devices used at trial sites can transfer data into systems independent of the site EHRs.

While it is theoretically possible to add trial sites to the data acquisition flow in each of these modalities the practical considerations, especially at-scale, make such universal implementations unfeasible. Therefore, the Logical Architecture must account for the fact that eSource data is acquired independent of trial sites while simultaneously recognizing that trial sites maintain certified copies of the data.

## Data Persistence & Computation Co-location.

eSource data from multivariate time-series digital signal acquisition can generate volumes surpassing the 10^12^ to 10^15^-byte range per study. Data volumes of this magnitude introduce technology challenges for clinical data persistence and the computational cycle bandwidth.

Architectural decisions that improve data access and availability drive efficient computational cycle use and improve corresponding insight yields . One straightforward decision in this regard is to co-locate eSource data with relevant computational environments. For example, splitting data into silos across multiple data management systems limits agility, forcing users to copy and re-aggregate data into usable forms before analysis.

## Data Clinical Relevance

eSource can generate data that lacks inferable *self-evident* clinical relevance. For instance, combined multivariate time-series digital telemetry -- e.g., accelerometer, meal log and environmental temperature – might correlate with and predict specific biological outcomes of interest; however, the individual digital signals possess no inherent clinical utility unless used as inputs to an algorithm that generates corresponding clinical measures.

This observation leads to a distinction between eSource data with intrinsic clinical relevance as a property at the time of its acquisition vs. data whose clinical relevance only manifests through algorithmic interpretation. eSource of the former category is termed *Clinically Relevant data* whereas data of the latter category is termed *Raw data*.

An important technology consideration of *Raw data* is the need for the application of algorithms – e.g., analytics – to transform it into *Clinically Relevant data.*

## Multi-Use

The velocity of eSource adoption is at least in part governed by the ease study teams can integrate eSource use into study protocols, and the associated risk-benefit tradeoff. Thus, technical solutions that reduce study team up front effort and implementation risk are favored. The easier the capability is to use, the more likely it will be used.

Architectures conceived to accommodate multiple studies are more likely to achieve ease-of-use from the study team perspective in that they provide well known logistical quantities such as service levels, time-to-readiness and standardized operational processes. Conversely, the effort and delivery risk inherent in designing, developing, testing and operating study-specific solutions may well outweigh any benefits from customized functionality.

Multi-use architectures must likewise strive to include the most general use cases. But solutions derived from such architectures need not implement all functionality before reaching an operational state. If the ability to scale across all use cases is preserved in solution instantiation, then organizations may flush out solutions with full architectural functionality later, or never, as needs merit.

## References

1. International Council for Harmonization (ICH). Integrated addendum to ICH E6(R1): Guideline for good Clinical Practice E6(R2). <https://www.ich.org/fileadmin/Public_Web_Site/ICH_Products/Guidelines/Efficacy/E6/E6_R2__Step_4_2016_1109.pdf>. Accessed July 2019.
2. European Medicines Agency. Reflection paper on expectations for electronic source data and data transcribed to electronic data collection tools in clinical trials. <https://www.ema.europa.eu/en/documents/regulatory-procedural-guideline/reflection-paper-expectations-electronic-source-data-data-transcribed-electronic-data-collection_en.pdf>. Accessed July 2019.
