# Supplementary material for: Technology Considerations for Enabling eSource in Clinical Research: Industry Perspective
Source: Ther Innov Regul Sci. 2020 Mar 11;54(5):1166–74. doi: 10.1007/s43441-020-00132-4 (PMC7458892; doi:10.1007/s43441-020-00132-4)
Supplement: Supplementary file 2 — Supplementary material 2 (DOCX 712 kb) [file 43441_2020_132_MOESM2_ESM.docx]

Technology Considerations for enabling eSource in Clinical Research:

Industry Perspective

# Appendix B: Patterns of Use

The four eSource modalities encapsulate multiple scenarios that eSource practitioners may put into current or future use. The commonalities between these scenarios give rise to twelve *patterns of use* that convey essential scenario attributes and simplify Stakeholder communication.

## EHR Modality

The four EHR patterns of use capture direct Sponsor interactions with institutional EHR systems and patients via personal health records (PHR), as well as indirect interactions via Health Information Exchanges (HIEs) that broker patient and institutional communication (see Diagram S1). Data acquisition supports communication protocols in use by the EHR, PHR and HIE systems. Acquired data is pseudo-anonymized to replace any patient-identifying values, normalized into a merged data set and persisted in preparation for analytic reduction and interpretation. Analysis results are exported into Sponsor data systems for incorporation into standard clinical data flow operations.


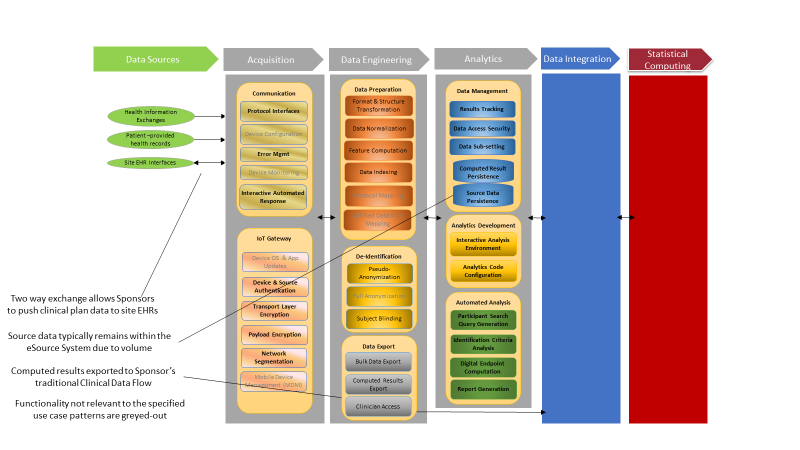


Diagram S1. Logical Architecture demonstrating EHR modality patterns of use.

**Pattern 1: EHR use in Clinical Trials.** This pattern captures direct site EHR to Sponsor clinical data exchange during clinical trials by eliminating double data entry. Many trial sites engage in point-of-care delivery to patients in addition to conducting clinical research, using their institutional EHR systems for both purposes. When conducting clinical research with Sponsors the site personnel often perform double data entry in the Sponsor-provided eDC system and institutional EHR system. This double data entry has the potential to cause undesirable side effects including:

- Non-value-added effort from site personnel performing redundant data entry,
- Manual transcription errors, and
- Delays in data availability and exchange between the trial sites and sponsors.

In some circumstances these delays incapacitate other high-value uses of the trial data; e.g., use of lab results to determine dynamic patient dose escalation decisions.

**Pattern 2: EHR use in Real World Evidence (RWE) Studies.** Clinical trials assess therapy efficacy and safety in controlled environments where clinicians must control for multiple health variables. The clinical conditions of therapy application can vary in the Real World (e.g., patient adherence) and thus affect therapy efficacy. Point-of-care health institutions gather and store tremendous quantities of Real-World data in their institutional EHR systems. The trove of data housed in these EHRs provide ideal input to RWE studies.

RWE study teams can in principle access EHR systems directly to extract data if appropriate security safeguards and patient informed consent are in place. A bigger potential technical issue arises from how to inform RWE study teams of relevant patient encounters at the institutions. Indirect use of EHR system data via 3^rd^ party Healthcare Information Exchanges (HIEs) and data brokers eliminate some of the issues with direct EHR access.

**Pattern 3: Protocol-based EHR programming.** A common clinical trial setup activity involves informing trial sites of the schedule of events for each patient’s site visit. Sponsors and sites engage in significant communication and sites manually enter the information into their EHR systems. As study protocols contain the visit structure and event activities it is possible to digitize this information and push it directly to site EHR systems, saving human effort and reducing errors due to manual data entry.

**Pattern 4: EHR Patient Recruitment.** Finding potential patient populations who match study inclusion/exclusion criteria is another significant study startup task. Site EHR systems contain information on large numbers of patients at the level of granularity required for inclusion/exclusion evaluation. Through use of appropriate patient identity and privacy safeguards, it is technically possible to examine site EHR records to find potential study candidates.

## Devices & Apps Modality

The four patterns of the Devices & Apps modality characterize data collection from, and direct interaction with, patients outside of traditional clinical trial site visit constraints (see Diagram S2). The potentially large number of devices necessitates use of Mobile Device Management (MDM) and IoT technologies in support of data acquisition. Acquired digital data is normalized, pseudo-anonymized (to replace any patient-identifying values) and persisted in preparation for analytic reduction and interpretation. Analysis results are exported into Sponsor data systems for incorporation into standard clinical data flow operations.


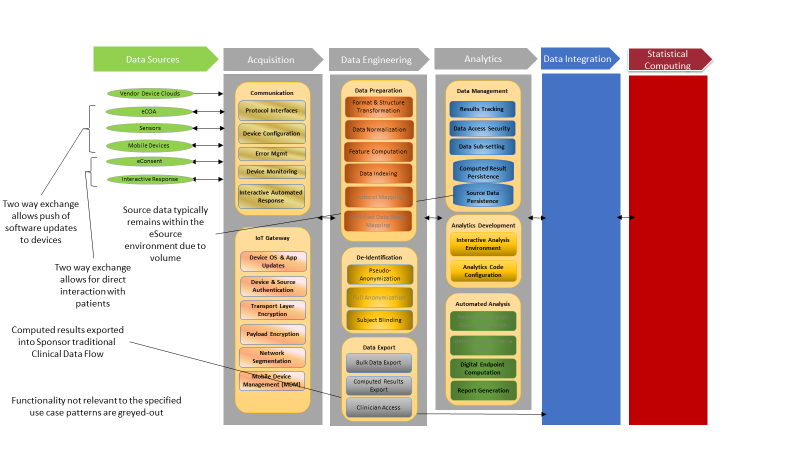


Diagram S2. Logical Architecture demonstrating Devices & Apps modality patterns of use.

**Pattern 5: eConsent.** Informed consent discussions typically occur in-person at trial sites. This necessitates patient travel and time, as well as site personnel bandwidth. Use of electronic informed consent (eConsent) techniques can be used in conjunction with existing site patient portals & EHR systems, or via mobile devices, to improve the patient experience and ease site burden.

**Pattern 6: App & Device Clouds.** Many existing and potential digital device offerings provide a cloud-based data aggregation point, alleviating the need for direct device communication. In such cases, it is possible to establish communication with the aggregation point and bypass direct device support.

Cloud-based data aggregations points offer convenience but are not necessary. If a device offering is not paired with such a cloud-based data aggregation point, then Pattern 7 (below) covers the use case.

**Pattern 7: Novel App & Devices.** When device manufacturer cloud-based aggregation points do not exist (e.g., experimental devices, mobile apps, custom sensors) the instantiated systems must implement direct communication with individual devices. In this pattern the systems must also handle device management tasks such as software upgrades.

**Pattern 8: Patient Registration and Device Setup.** In several device provisioning situations patients receive devices for clinical data capture via postal delivery and perform activation in their home. This is true when the device setup process is straightforward and/or when patients live far away from trial sites; e.g., in rural areas. Automated device setup facilitates activities such as association between devices and patients to occur on the Sponsor backend Interactive Web Response Systems (IWRS) without direct clinical site support.

## Non-CRF Modality

**Pattern 9: Non-CRF Data Source Exchange**. Non-CRF data generated by 3^rd^ parities (e.g., central labs) flows into Sponsor data systems via automated data exchange based upon system-to-system integrations. While this pattern stands alone within the Non-CRF modality, it represents a significant use case for most Sponsor clinical data flows (see Diagram S3).


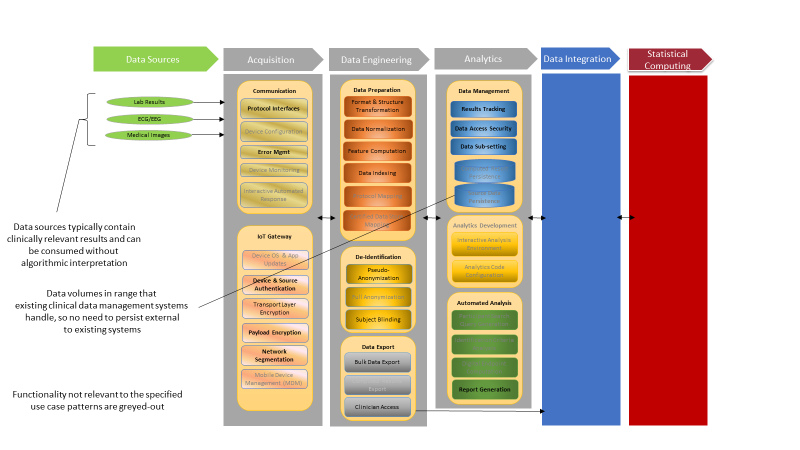


Diagram S3. Logical architecture demonstrating the Non-CRF modality pattern of use.

This pattern supports automated, high throughput data acquisition from systems such as medical data instruments. Incoming data is indexed, transformed, and pseudo-anonymized (to remove any patient identifying information) for ingestion by Sponsor data systems.

## Direct Data Capture Modality

**Pattern 10: Capture of CRF Data Using Mobile Devices**. Collection of CRF data at trial sites using mobile devices has the potential to improve site personnel efficiency and reduce transcription errors. In such situations, site staff enter relevant healthcare information directly into electronic tablets during patent visits rather than manually transcribing data into eDC systems post-visit. This eliminates the need for clinicians to enter data twice – once into the site data system (e.g., EHR) and again into the Sponsor eDC.

If the collected data only pertains to the clinical trial – e.g., rating scales -- the data flow is straightforward. If the CRF-mandated data values collected by the mobile devices coincide with patient medical care needs then the data flow is more complex, as these values must be transmitted into the site EHR. The use of Direct Data Capture (DDC) should not limit the capability of clinicians to record and maintain non-protocol mandated information^11^(see Diagram S4). Therefore, this pattern incurs the potential burden of validated interoperability with each site EHR.


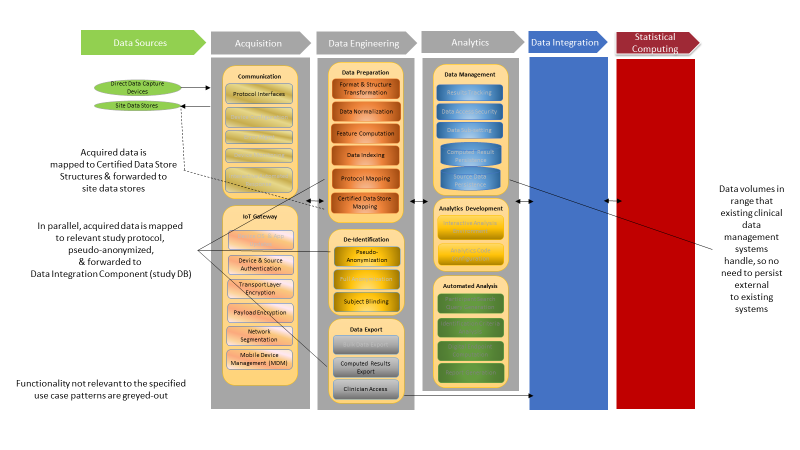


Diagram S4. Logical architecture demonstrating the Direct Data Capture pattern of use.

This pattern acquires data from DDC devices, maps/filters values to the relevant study protocol, performs patient identity pseudo-anonymization and forwards the values to the Sponsor clinical data flow. In parallel, data values are transformed into consumable structures for the site data systems (e.g., EHR) and forwarded to the site for ingest. One suggested alternate pattern forwards data to the site data store and waits for an acknowledgement before filtering/mapping and de-identifying the data values for Sponsor consumption^12^.

## Other Patterns

These patterns acquire existing digitized clinical records from multiple sources (e.g., completed studies, institutional EHR records, public and purchased data sets), transform and normalize the records into a merged data set, anonymize patient identifying data and make the it available for analysis. The analysis environment provides efficient data set access, unlimited compute cycles, and access to commonly used analytics tools.

**Pattern 11: Exploratory & Analytics Development**. A key eSource goal involves finding correlations between digital signals and biological events, then encapsulating the correlations into validated predictive analytics. The process requires aggregation and analysis of data across many studies and ancillary sources that often occur outside specific study execution. The Logical Architecture provides a common environment where Big Data-scale data reside alongside scalable compute environments and analysis tools to facilitate analytics research (see Diagram S5). Any predictive analytics developed within this pattern are used as input to subsequent clinical trials or digitally-enhanced therapies.


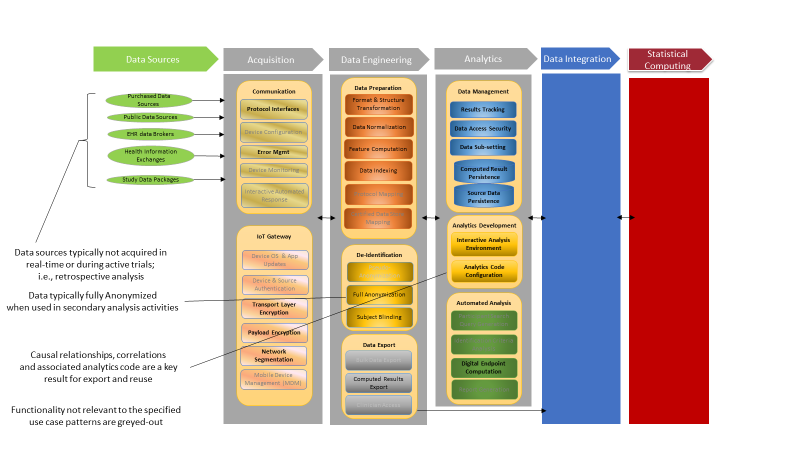


Diagram S5. Logical architecture demonstrating the Exploratory & Analytics Development and Synthetic Control Arm patterns.

**Pattern 12: Synthetic Control Arms**. Synthetic control arms are an emerging method for modeling study placebo arms with previously collected information, instead of running active trial control arms with patients receiving a placebo (see Diagram S5). Where deemed appropriate, this approach provides several significant advantages to patient care and study efficiency^1,2^. The pattern applies machine learning techniques to match previous patient data to the target study criteria (e.g., eligibility criteria, biometric covariates), model the outcome, and export the model results to existing Sponsor data systems for more traditional study analysis.

## References

1. Synthetic Control Arm: The End of Placebos? Abbvie News. <https://stories.abbvie.com/stories/synthetic-control-arm-end-placebos.htm>. Accessed July 2019.
2. Donald A. Berry, Michael Elashoff, Steven Blotner, et al. Creating a synthetic control arm for previous clinical trials: Application to establishing early end points as indicators for overall survival in acute myeloid leukemia. Journal of Clinical Oncology 2017 35:15_suppl, 7021-7021.
