# Supplementary material for: Technology Considerations for Enabling eSource in Clinical Research: Industry Perspective
Source: Ther Innov Regul Sci. 2020 Mar 11;54(5):1166–74. doi: 10.1007/s43441-020-00132-4 (PMC7458892; doi:10.1007/s43441-020-00132-4)
Supplement: Supplementary file 3 — Supplementary material 3 (DOCX 34 kb) [file 43441_2020_132_MOESM3_ESM.docx]

Technology Considerations for enabling eSource in Clinical Research:

Industry Perspective

# Appendix C. Logical Architecture Implications.

The Logical Architecture assertions have implications for the axioms on which clinical research currently operates. These implications could affect how Stakeholders utilize eSource technologies or, conversely, how the axioms of clinical research evolve to meet the implications. The topics below illustrate some potential implications and suggest how Stakeholders might respond to the challenge. Note that suggested responses do not constitute official guidance and are only given to stimulate thought within the community.

**Acquiring data across national boundaries**. The networked nature of digital data acquisition and persistence may work best, from a technology perspective, when implemented as sets of centralized aggregation points. This creates potential situations where data is gathered from patients or sites in country A from a centralized system physically instantiated in country B.

Accounting for regional differences in data privacy laws and regulatory authority requirements is not a new consideration in clinical trial design, especially for large trials that utilize many trial sites; however, the constraints typically impact procedural aspects of trial operations rather than the acquisition of data. Implications now exist for device-network connectivity as a function of national boundary, as well as where clinical trial Sponsors choose to implement data aggregation points. Even when clinical research Sponsors carefully plan for device-network and aggregation point considerations, situations can occur where patients travel from one country to another and their connected devices continue to stream telemetry to the local network.

**Adverse Events**. Digital devices can generate constant and voluminous streams of patient health telemetry. Practical considerations preclude active and continuous data monitoring by HCPs, especially when patients reside outside healthcare institutions. If telemetry should indicate a patient Adverse Event (AE) or other health issue, will the system acquiring the telemetry be able to detect it? And if so, how will the acquiring system notify HCPs to intervene?

In principle, automated agents can continuously monitor near real-time telemetry streams to detect events if the prerequisite analytics are developed and validated. Likewise, HCPs can be notified by the automated agents if a detection occurs. Implementing such a scheme requires Stakeholders to invest in establishing the analytics and associated near real-time data processing capabilities, create applicable regulatory guidance and staff trials with enough HCP bandwidth to handle the additional care needs.

Hypothetically, detecting and addressing such adverse events in study participants could end up obfuscating serious adverse events; e.g., analytics detect adverse event X in the telemetry stream, HCPs quickly intervene with the affected patients, and quick action keeps serious adverse event Y from occurring. The moral and regulatory implications of this hypothetical situation should be duly considered by all Stakeholders to assess how it might affect future study designs.

**Data Review.** Good Clinical Practice implementations typically call for Sponsors to review and scrutinize data for inconsistences (e.g., values out of expected range) as it flows from trial sites, and to interact with trial sites for clarification/correction of unexpected data values. Data corrections are rigorously documented, and the resulting audit trails used to assure data integrity.

This established data review practice often stems from the need to catch potential errors introduced during transcription of trial site data into EDC systems. eSource eliminates manual transcription, thus removing practical need for the established data review practice. For the Devices & Apps modality the practice becomes a practical impossibility due to the data volumes and complexities of digital signal inspection.

Data errors and inconsistences can still exist in eSource data – e.g., mis-mapping of EHR data values into Sponsor data systems, digital device glitches, central lab data transmission errors – but the ability to detect and correct such errors likely requires an automated, algorithmic approach to data interpretation; i.e., data review becomes a *Data Engineering* activity. This shift has implications to existing Sponsor clinical data flow processes, regulatory guidance and providers of eSource technology solutions.

**Analytics validation**. The data volume and complexity arising in the Devices & Apps and EHR modalities can necessitate use of automated analytics to summarize and process the acquired data. These analytics, especially those used to correlate between digital signals and biological events, occupy a new niche in the clinical data flow from existing statistical techniques; i.e., these analytics can affect trial operations as well as decisions made by HCPs in patient care.

Stakeholders must consider the conditions under which these analytics should be employed, what it means to validate analytics functionality based upon intended use, and how regulatory authorities align to the clinical relevance of computed analytic results. For instance, if input data contains missing values then under what circumstances can analytics use imputation to fill in the gaps, and what level of software testing is enough to cover all reasonable contingencies?

**Data validation and integrity**. The nature of digital data acquisition and transformation introduces complexities into traditional clinical research data quality safeguards. These complexities generally arise from data interoperability nuances between systems, data security and eSource data volumes. Four specific use cases are presented below.

- Data stored in site EHRs is not necessarily collected solely for clinical research purposes. Issues that do not affect data usability at individual healthcare institutions can become problematic when combined across institutions for study use; e.g., use of varying coding standards for lab tests, emphasis on data field completeness, key information captured in free-text fields.
- Sponsor access to site EHR data necessitates implementation of safeguards that assure Sponsor only glean patient data for which approved study protocols mandate. Stakeholders must agree upon best practices for exposing patient data, authenticating and authorizing access, filtering data according to study protocols, and assuring patient identity pseudo-anonymization.
- Enabling analysis agility, by tweaking inputs to deal with data inconsistencies traditionally remedied in the data review process prior to statistical analysis, implies rapid changes to transformations and/or use of on-the-fly imputation. This potentially conflicts with principles of system validation, where functionality is rigorously tested and approved prior to production use. Stakeholders must align upon what flexibility, if any, might be allowed in the data engineering process for quick response to unforeseen circumstances; e.g., reliance upon professional judgement by highly trained personnel to make changes to the clinical data flow when such changes are flagged for review and approval post-change.
- Anecdotal stories of people attaching mobile health devices to pets for generation of fake physical activity data, or patients filling out weeks of eCOA diary entries in site parking lots, highlight the issue of device data lineage. How do sites and Sponsors assure that device and sensor data originate from patients in the manor intended? A straightforward way to remedy this problem involves building fraud detection capabilities into device and eSource solutions, but requires Stakeholder partnership with suppliers to influence product offerings.

**HCP access to digital data streams**. Devices & Apps data streams provide a potentially useful monitoring capability for use in patient care, but the quantity and complexity of data from digital devices makes it unsuitable for direct HCP use. In many cases this data may be summarized and made accessible via intuitive user interfaces, allowing HCPs to quickly assess patient status. Therefore, technology instantiations should provide portals where device telemetry data is summarized and “dashboarded” for HCP consumption. However, the potential utility of summarized telemetry stream monitoring should be balanced with limiting HCP burden during trial execution.

**Systems of record**. With use of eSource trial sites do not necessarily acquire the digital data. This contrasts with main-line clinical research where data chain-of-custody is straightforward to establish; trial sites acquire the data and, therefore, retain the certified systems-of-record. When the data is not acquired by the trial site the system-of-record becomes more complex to establish.

Potential solutions to this conundrum exist, but widely understood regulatory guidance covering all scenarios remains in flux. The US FDA states that clinical digital data sources that do not require site HCP review for patient safety and efficacy may transfer directly into Sponsor data systems, and in these cases the Sponsor retains the system of record ^1^. Related EMEA guidance states that use of eSource “*must not impoverish clinical care by depleting the medical records or limiting the capability of the healthcare professional to record, maintain and trace non-protocol mandated information^2^*”.

The current least-risk mitigation path is to use services of a 3^rd^ party to host the eSource data repository system-of-record. In this way the 3^rd^ party acts on behalf of trial sites, alleviating the need for each site to acquire and maintain the eSource data – if the study protocol does not mandate investigator use and review of the data for patient care.

**Primary Investigator signoff**. In main-line clinical trials that use EDC systems for data acquisition the process of site Primary Investigator (PI) data approval is a straightforward activity; i.e., the PI reviews and electronically signs each eCRF sent to the Sponsor to assure its validity. As eSource data does not flow through an EDC system, the existing process of PI data approval cannot be duplicated. Perhaps more importantly, what is the meaning of PI review and approval of data not acquired by devices under site control?

Initial US FDA thoughts on this topic^15^ indicate that data transmitted directly to Sponsor data systems, especially large volume data, does not need to be reviewed by site PIs. Data specified by study protocols as captured in eCRFs is definitely in scope for PI signoff, but data from other sources are open to negotiation^1^. While formal regulatory guidance on this topic should eventually focus on this topic, the initial FDA position provides directional clarity on how eSource technology solutions can address the need.

## References

1. CDER Health IT Board: SCDM eSource Project Meeting. R. Rocca et al. March 12, 2019, 1:00pm-2:30pm EST. FDA WO-BLDG 32 ROOM 1305, 10903 New Hampshire Ave, Silver Spring, MD 20993.

1. [eSource Direct Data Capture (DDC) Qualification Opinion](https://www.ema.europa.eu/en/documents/regulatory-procedural-guideline/esource-direct-data-capture-ddc-qualification-opinion_en.pdf). European Medicines Agency. <https://www.ema.europa.eu/en/documents/regulatory-procedural-guideline/esource-direct-data-capture-ddc-qualification-opinion_en.pdf>. Accessed July 2019.
